# Exploring the pathogenesis of MAFLD from an immunological perspective: from the perspective of the cGAS/STING/NF-κB signaling pathway

**DOI:** 10.3389/fimmu.2025.1674018

**Published:** 2025-09-29

**Authors:** Ruiyuan Tian, Yong Li

**Affiliations:** ^1^ Department of Gastroenterology, Qilu Hospital of Shandong University, Jinan, China; ^2^ Department of Hepatobiliary Diseases, Affiliated Hospital of Shandong University of Traditional Chinese Medicine, Jinan, China

**Keywords:** metabolically associated fatty liver disease, immune signaling pathway, cGAS/STING/NF-κB, pathogenesis, intervention strategy

## Abstract

Metabolic-Associated Fatty Liver Disease (MAFLD) is the most common cause of chronic liver disease and also a major contributor to liver disease-related complications and mortality. It is closely associated with cardiovascular disease (CVD), stroke, type 2 diabetes mellitus (T2DM), chronic kidney disease (CKD), and non-liver tumors, and has become a significant global public health issue. In recent years, studies have respectively revealed the relationships between the cGAS/STING and STING/NF-κB signaling pathways and MAFLD. Although, in addition to cyclic GMP-AMP synthase (cGAS), various other DNA sensors can also recognize DNA molecules and activate stimulator of interferon genes (STING), their localization response capability and hepatocyte targeting are relatively weak, and most of them only function in specific cell types or physiological states. As a key innate immune mediator, cGAS is the core molecule that activates the classical STING pathway. Therefore, the cGAS/STING/NF-κB signaling pathway may form an important pathological chain of “DNA stress - inflammation - metabolic abnormality” in MAFLD. Consequently, it is necessary to explore the mechanism of action and research progress of the cGAS/STING/NF-κB signaling pathway in MAFLD, which provides new insights for the mechanism research and treatment of MAFLD.

## 1 Introduction

MAFLD is a metabolic disorder-related liver disease closely associated with insulin resistance (IR) and overnutrition in genetically susceptible individuals. It encompasses non-alcoholic fatty liver disease (NAFLD), mixed fatty liver disease (FLD) with coexisting etiologies, and may occur in patients with other liver diseases such as chronic hepatitis B or autoimmune liver disease. Epidemiological studies indicate that with the prevalence of metabolic disorders like obesity and diabetes, the global prevalence of MAFLD has been increasing annually, establishing it as one of the most common liver diseases worldwide. Research has shown that the burden of non-alcoholic fatty liver disease is rapidly escalating globally (1). The diagnosis of MAFLD requires at least one metabolic risk factor, such as obesity, T2DM, hypertension, or dyslipidemia (2, 3), with over 95% of NAFLD patients meeting the diagnostic criteria for MAFLD (4). Currently, MAFLD has a global prevalence of approximately 38% (5), with a prevalence of 50.7% (95% CI, 46.9–54.4) in overweight or obese adults. Men exhibit a higher prevalence of MAFLD (59.0%; 95% CI, 52.0–65.6) compared to women (47.5%; 95% CI, 40.7–54.5) (6). The global prevalence of NAFLD/MAFLD in T2DM patients is 65.33% (95% CI, 62.35%–68.18%) (7, 8), while the estimated global prevalence in children and adolescents is 7.4% (9). Notably, MAFLD is not confined to obese or overweight individuals; individuals with normal body weight are also at risk (10–12). The incidence of NAFLD in non-obese or lean populations is 24.6 (95% CI 13.4–39.2) per 1000 person-years (13). In multi-ethnic populations in the United States, the prevalence of non-obese NAFLD is 9.6%, with one-third comprising elderly individuals, men, and others, and a higher mortality rate compared to obese NAFLD (14). Lean NAFLD is defined as “metabolically unhealthy normal weight,” characterized by reduced subcutaneous fat and ectopic hepatic fat deposition (15).

MAFLD is associated with a variety of systemic diseases. Owing to its close association with insulin resistance—which constitutes a major cardiovascular risk factor in non-diabetic patients (16)—studies have demonstrated (17–20) that individuals with MAFLD exhibit a higher cumulative incidence and risk of cardiovascular diseases (including hypertension and atherosclerosis) compared to those without MAFLD (21, 22), with an associated hazard ratio (HR) of 1.38 (95% confidence interval: 1.37–1.39); the HR for stroke is 1.55 (95% confidence interval: 1.37–1.73).The prevalence of CKD related to non-alcoholic fatty liver disease ranges from 20% to 55%, and its severity is correlated with CKD staging (23–25). Furthermore, the risk of developing CKD in patients with MAFLD/MASLD increases with the severity of fatty liver. Even after disease remission, patients with a history of moderate-to-severe fatty liver still have a relatively high risk of CKD. In addition, the progression from simple steatosis to non-alcoholic steatohepatitis (NASH) is associated with an increased incidence of liver cirrhosis and hepatocellular carcinoma (26), as well as a higher overall mortality rate (27). This underscores the multifaceted impacts of MAFLD and its potential threat to human health.

Lean NAFLD exhibits histological phenotypes similar to those in obese patients, carrying a greater risk of severe liver disease progression, with higher prevalence of diabetes, advanced fibrosis, and cirrhosis. It is characterized by impaired glucose metabolism, adipose tissue dysfunction, and a high proportion of lobular inflammation (28, 29). Consequently, lean NAFLD is associated with a higher degree of visceral adipose tissue and adipose tissue dysfunction, with the severity of histological injury independent of body mass index (30).

As a prevalent chronic liver disease worldwide, MAFLD imposes a significant public health burden due to its potential complications, and no effective treatments currently exist (31). Its pathogenesis and progression are regulated by factors such as hepatic lipid accumulation, oxidative stress, insulin resistance, immune and metabolic dysfunction, and apoptosis (32–34). Epidemiological features reflect its complexity, closely intertwined with immune, inflammatory, and metabolic dysfunction, involving interactions across multiple biological processes and signaling pathways. The cGAS/STING pathway has been extensively studied in autophagy, infection, metabolism, cancer, inflammation, and aging (35), but the mechanisms underlying cGAS/STING/NF-κB interactions in MAFLD pathogenesis remain underexplored. This pathway may play a complex role in MAFLD, making it crucial to investigate its mechanisms to identify novel therapeutic targets.

## 2 The cGAS/STING/NF-κB signaling pathway and MAFLD

The immune system is intimately linked to metabolic regulation across all animal species. Beyond its primary role in innate defense against pathogen infections, research has revealed that the cGAS-stimulated pathway plays a pivotal role in metabolic processes. The cGAS-STING axis serves as a central innate immune defense mechanism against viral infections, primarily driving the production of type I interferons. This pathway is not only critical for antiviral responses but also influences bacterial infection defenses, immune-mediated diseases, and inflammatory processes (36, 37). Immune responses and metabolic homeostasis are deeply intertwined, with chronic low-grade inflammation known to promote the development of metabolic disorders (38–40). Over the past decade, growing evidence has highlighted the significance of innate immunity in the pathogenesis of hepatic steatosis and NAFLD (41).

Existing studies have shown that in addition to the classical pathway, a variety of DNA sensors can also activate the STING signal, including IFI16, DDX41, DAI, MRE11, and DNA-PK. These sensors together form a multi-dimensional regulatory network for STING activation through methods such as directly recognizing DNA molecules, regulating cGAS activity, or mediating non-classical signaling pathways. Among them, cGAS, as a core molecule, dominates pathway activation, while IFI16, DDX41, DAI, MRE11, and DNA-PK play supplementary regulatory roles in specific cell types or physiological conditions. The dynamic balance of the aforementioned mechanisms is of great significance in tumorigenesis, infection response, and the progression of autoimmune diseases.

Specifically, interferon-inducible factor 16 (IFI16) is crucial for maintaining STING protein levels and the immune response after activation of the IFN-γ response pathway in metastatic melanoma (42); cell cycle inhibitors can mediate anti-tumor immune responses in liver cancer by activating the hypoxia-induced DDX41/STING pathway (43); DAI (also known as DLM-1/ZBP1), as a cytoplasmic DNA sensor, is a key activator of innate immune responses (44), although studies have found that mice with DAI gene knockout can still express large amounts of type I interferons under stimulation with type B DNA (45); MRE11 can participate in pathway regulation by releasing cGAS from nucleosome sequestration during tumorigenesis (46), and its dependent instability in mitochondrial DNA fork protection can activate the cGAS immune signaling pathway (47); meanwhile, as a DNA damage sensor, MRE11 can also recognize cytoplasmic double-stranded DNA (dsDNA) and induce type I interferons by regulating STING transport (48); DNA-PK, as a DNA sensor for IRF-3-dependent innate immunity (49), can activate STING in DNA damage response through a cGAS-independent pathway (50).

**Figure 1:**
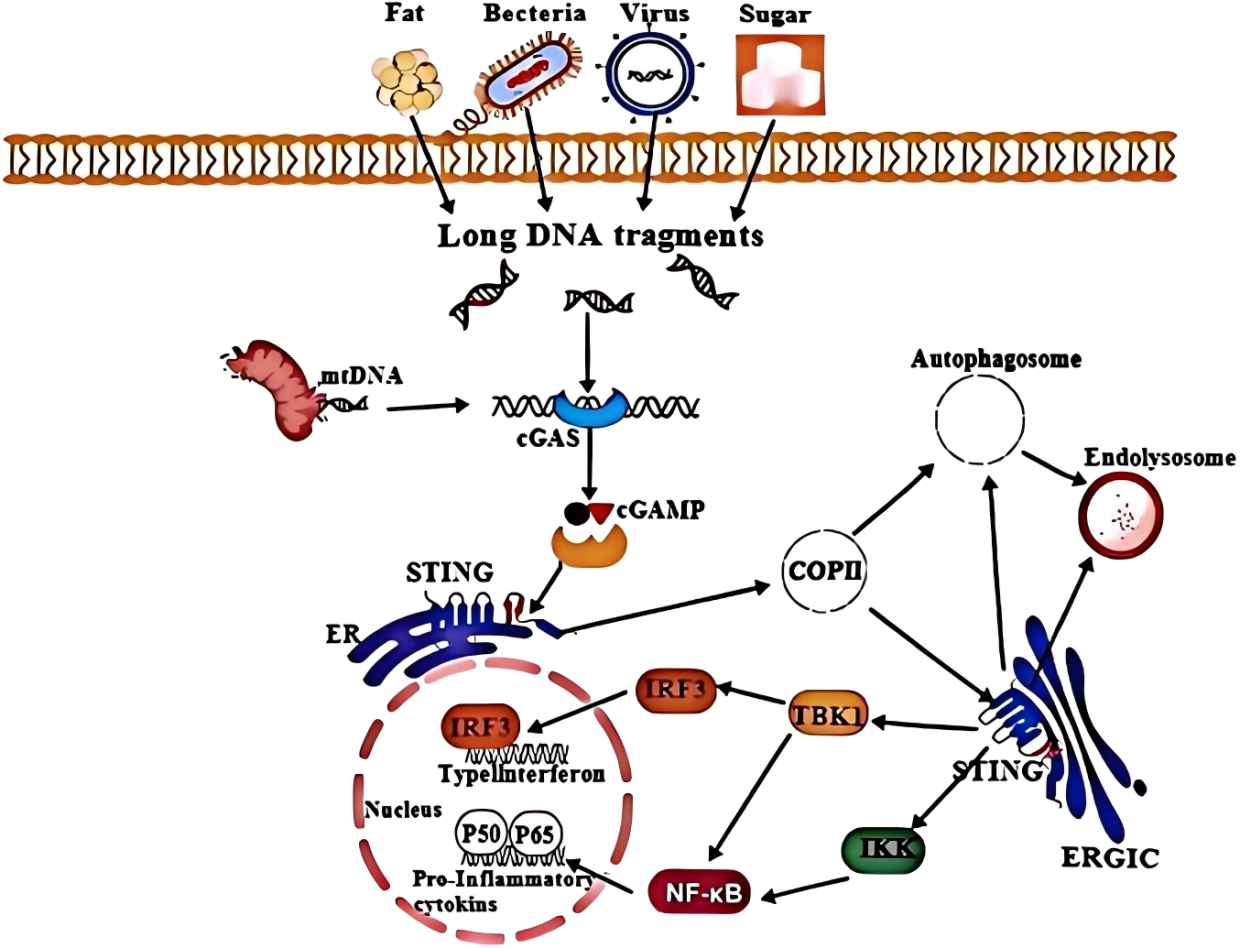
cGAS-STING signaling pathway. When bacteria and viruses invade, or high sugar is ingested, or lipid accumulation occurs in liver cells, or when cells are damaged (including damaged mtDNA), DNA fragments accumulate in the cytoplasm. The cGAS recognizes cytoplasmic long DNA fragments and catalyzes the synthesis of 2 ′ 3 ′ - cyclic GMP cAMP (cGAMP). CGAMP binds to the dimer of STING located on the endoplasmic reticulum (ER) membrane, causing oligomerization of STING. This leads to the incorporation of STING into the shell protein complex II (COPII) vesicles, initiating the transport of STING from the ER to the Golgi apparatus and the ER Golgi apparatus. STING activates serine/TBK1, which in turn phosphorylates IRF3. Phosphorylated IRF3 then activates the expression of IFN-I in the nucleus. The activation of STING also leads to the activation of NF-κB and the formation of autophagosomes through non classical mechanisms. NF-κB activation promotes the expression of pro-inflammatory cytokines, ultimately leading to the degradation of STING in autophagosomes and lysosomes.

Studies have shown that high glucose levels, intracellular lipid accumulation in hepatocytes, activation of dsDNA and cyclic dinucleotides (CDNs), endoplasmic reticulum stress (ER stress), mitochondrial stress, as well as energy imbalance in metabolic cells and immune cells, can activate the cGAS-STING pathway. STING is relocalized from the endoplasmic reticulum to the endoplasmic reticulum-Golgi intermediate compartment, leading to the palmitoylation of cysteine residues at positions 88 and 91 in the N-terminal domain of STING in the Golgi apparatus, thereby activating TANK-binding kinase 1 (TBK1). TBK1 phosphorylates the serine and threonine residues of STING, and the phosphorylated STING has increased affinity for IRFs, thus recruiting interferon regulatory factor 3 (IRF3) for TBK1-dependent phosphorylation and activation. The activated IRF3 dimers translocate to the nucleus, inducing the expression of type I interferons (IFNs) and various immunoregulatory factors, thereby triggering pro-inflammatory responses and metabolic disorders (51). Pathological overactivation of this pathway is closely linked to metabolic conditions such as obesity, NAFLD, insulin resistance, and cardiovascular diseases (CVDs). Research has identified interactions between cGAS-STING and other signaling pathways—including nuclear factor-κB (NF-κB), Jun N-terminal kinase (JNK), AMP-activated protein kinase (AMPK), mammalian target of rapamycin (mTOR), lipophagy, pyroptosis, and the insulin signaling cascade—as key mechanisms through which cGAS-STING modulates inflammation and metabolic homeostasis (38, 39). (**Figure 1**).

In MAFLD, intracellular environmental changes driven by factors like fat accumulation lead to abnormal DNA release and recognition (52). These changes induce chromatin modifications, exposing previously sequestered DNA and activating cGAS. cGAS catalyzes the synthesis of the second messenger cGAMP, which is transported to STING on the endoplasmic reticulum membrane (53), thereby initiating the cGAS-STING innate immune pathway. Activated STING triggers NF-κB, causing inflammatory responses and metabolic disorders, thereby promoting the progression of MAFLD (54). Recent studies have shown that the NLRP3 inflammasome can be directly activated by cGAMP or STING, which can enhance hepatocyte pyroptosis, inflammation, and fibrosis. This may be an important mechanism that exacerbates MAFLD fibrosis (55, 56).

### 2.1 cGAS - STING pathway

#### 2.1.1 cGAS-STING pathway and macrophages in liver inflammation and lipid accumulation

Tightly associated with nucleosomes, it acts as a mediator regulating innate immunity, capable of recognizing DNA abnormally present in the cytoplasm and can be activated by double-stranded dsDNA from multiple sources (58, 59). Under physiological conditions, cGAS remains in an inactive state. Under pathological conditions, once activated, cGAS catalyzes the synthesis of the second messenger cGAMP. This activates stimulator of STING, inducing its conformational change and translocation from the endoplasmic reticulum to the Golgi apparatus, which then recruits and activates downstream signaling molecules to transmit immune responses (60).

Liver macrophages exhibit heterogeneity in their origins, including Kupffer cells derived from the yolk sac/fetal liver and populations derived from bone marrow monocytes. STING is a transmembrane protein primarily localized in the endoplasmic reticulum; it is mainly expressed in liver macrophages, including CCR2+, S100A9+, Kupffer cells, and CD163+ cells (61, 62). Conformational changes in STING drive the production of factors that promote lipid accumulation in hepatocytes and pro-inflammatory responses, thereby contributing to hepatic steatosis, inflammation, and fibrosis (63).

Therefore, in recent years, the role of the cGAS-STING pathway in alcoholic/non-alcoholic steatohepatitis has received widespread attention (58). Non-alcoholic steatohepatitis is considered to be associated with sterile inflammation mediated by innate immunity; in the pathogenesis of NAFLD (non-alcoholic fatty liver disease), the involvement of cGAS-STING signaling via DNA-induced type I interferon responses has become increasingly prominent (64, 65). Endoplasmic reticulum stress in MAFLD (metabolic-associated fatty liver disease) activates the unfolded protein response (UPR), upregulates the expression of STING, mediates immune and metabolic responses, and exacerbates hepatic inflammation and cellular damage (66, 67). Excessive activation of cGAS-STING can also aggravate chronic inflammation, leading to metabolic dysfunction, aging, and conditions such as obesity and neurodegenerative diseases (68).

Recent studies have emphasized that, beyond the traditional M1/M2 model, macrophages also exhibit a series of activation states, which influence the diversity of their functions (69). Under cGAS-STING activation, macrophages differentiate into pro-inflammatory M1 phenotypes, secreting tumor necrosis factor-α (TNF-α) and interleukin-1β (IL-1β), and Interleukin-6(IL-6) to induce hepatic inflammation and fat deposition, accelerating NAFLD progression (68). cGAS can also polarize macrophages to an M1 phenotype via the mTORC1 pathway, mediating inflammatory responses (70). Importantly, macrophage - mediated insulin resistance exists independently of inflammation and appears before the onset of inflammation (71). Therefore, in the early stage of a high - fat diet without inflammation, depleting liver macrophages can improve insulin sensitivity (72, 73). Pro-inflammatory cytokines further inhibit hepatocyte lipid metabolism genes via the PPAR-α pathway, promoting steatosis (12, 74). In lean NAFLD mice, abnormal elevation of the macrophage cholesterol sensor SCAP drives STING translocation to the Golgi apparatus, recruiting TBK1 for phosphorylation, which induces metabolic inflammation, promotes lipolysis, and increases hepatic lipid deposition (75).

STING activation in lipid metabolism pathways can exacerbate fat accumulation (76). Excessive hepatic lipid storage generates lipotoxins, inducing mitochondrial dysfunction, endoplasmic reticulum stress, and reactive oxygen species (ROS) overproduction (64, 65). Lipotoxicity further triggers the release of mitochondrial DNA, lipid antigens, and adipokines, reactivating cGAS-STING to form a self-reinforcing cycle of inflammation and metabolic dysregulation.

#### 2.1.2 cGAS-STING pathway is activated by endoplasmic reticulum/mitochondrial stress

This chronic inflammation caused by glucotoxicity and resulting in IR is also common to NAFLD and T2DM. Hyperglycemia causes abnormal intracellular protein glycosylation, leading to misfolded or unfolded proteins accumulating in the endoplasmic reticulum and triggering the unfolded protein response (78). Some studies have found that oxidative stress induced by hyperglycemia promotes mitochondrial dysfunction and the release of mitochondrial DNA, which subsequently activates the cGAS-STING pathway as well as cGAS-STING-dependent IRF3 (79). It also induces the polarization of macrophages into a pro-inflammatory phenotype and the release of pro-inflammatory cytokines (80). A large number of studies have shown that in mammals, endoplasmic reticulum stress is closely related to the regulation of nutrient sensing and glycolipid metabolism. Endoplasmic reticulum stress affects the regulatory mechanisms of metabolic pathways in different tissues and organs such as the liver, adipose tissue, islets, and hypothalamus, thus playing an important role in the occurrence and development of glycolipid metabolism disorders.

#### 2.1.3 High-fat diet, gut dysbiosis, and cGAS activation in MAFLD

Increasing evidence highlights a close association between the gut microbiota and NAFLD progression (82), with microbial imbalance identified as a key determinant in MAFLD and metabolic syndrome (83). Diet acts as a powerful modulator of gut microbial composition and mucosal immune responses (84, 85).

High-energy diets and overnutrition are major factors altering fat metabolism, systemic inflammation, and gut microbiota profiles (86). A high-fat diet can trigger cGAS-STING-IRF3-mediated inflammatory responses (87), while diets rich in animal protein promote anti-inflammatory macrophage activity, worsen intestinal inflammatory damage, and rapidly reshape gut microbial communities (88). Consuming a high-sugar diet (HSD) impairs the intestinal mucosal barrier and modulates immune responses (89); excessive dietary monosaccharides reduce microbial diversity and deplete short-chain fatty acids (SCFAs), which possess immunomodulatory properties and influence colonic regulatory T cell absorption and macrophage antibacterial activity (90). SCFA depletion alters both intestinal bacterial and fungal microbiota composition. Gut microbiota-derived endotoxins like lipopolysaccharide (LPS) enter the liver via the portal vein, activating hepatic Toll-like receptor 4 (TLR4). In LPS-induced inflammation, cGAS is upregulated through the TLR4 pathway (91), subsequently activating the STING-NF-κB signaling cascade and inducing hepatic inflammatory responses.

#### 2.1.4 cGAS-STING pathway promotes hepatic fibrosis and hepatic sinusoidal microthrombosis

Activation of this signaling cascade triggers the release of pro-inflammatory factors, which not only recruit immune cell infiltration but also amplify local inflammatory responses. When hepatic sinusoidal endothelial cells are damaged or inflamed, conditions conducive to microthrombus formation arise, accompanied by endothelial-mesenchymal transition (EndMT), directly contributing to microthrombus development (93).

Upregulated cGAS-STING signaling initiates signals that drive the activation of hepatic stellate cells (HSC). The resulting cytokines and chemokines act on HSC, inducing the expression of fibrosis-related genes like α-smooth muscle actin (α-SMA) and collagen-I, thereby exacerbating liver fibrosis and intrahepatic inflammation. Additionally, this pathway promotes the upregulation of differentially expressed genes (DEG) in liver tissue, inducing phenotypic changes in hepatic sinusoidal endothelial cells that enhance inflammatory responses and release pro-coagulant factors, facilitating microthrombus formation. These phenotypic alterations lead to reduced or absent fenestrae, increased resistance to sinusoidal blood flow, and impaired circulation—mechanisms critical for both thrombus formation and the development of portal hypertension (94, 95).

#### 2.1.5 The cGAS-STING signaling pathway and renal injury

The cGAS-STING signaling pathway plays a key role not only in innate immune surveillance but also in additional biological processes, including kidney diseases (96, 97). MAFLD serves as a significant risk factor for CKD (98, 99). Recent in-depth investigations into the innate immune functions of this pathway have revealed emerging evidence that it may possess roles beyond traditional immune surveillance. Consistent with this, dysregulation of cGAS-STING signaling in adipocytes, hepatocytes, and renal proximal tubular epithelial cells has been linked to metabolic dysfunction, energy balance disorders, and kidney pathologies (100, 101).

**Figure 2:**
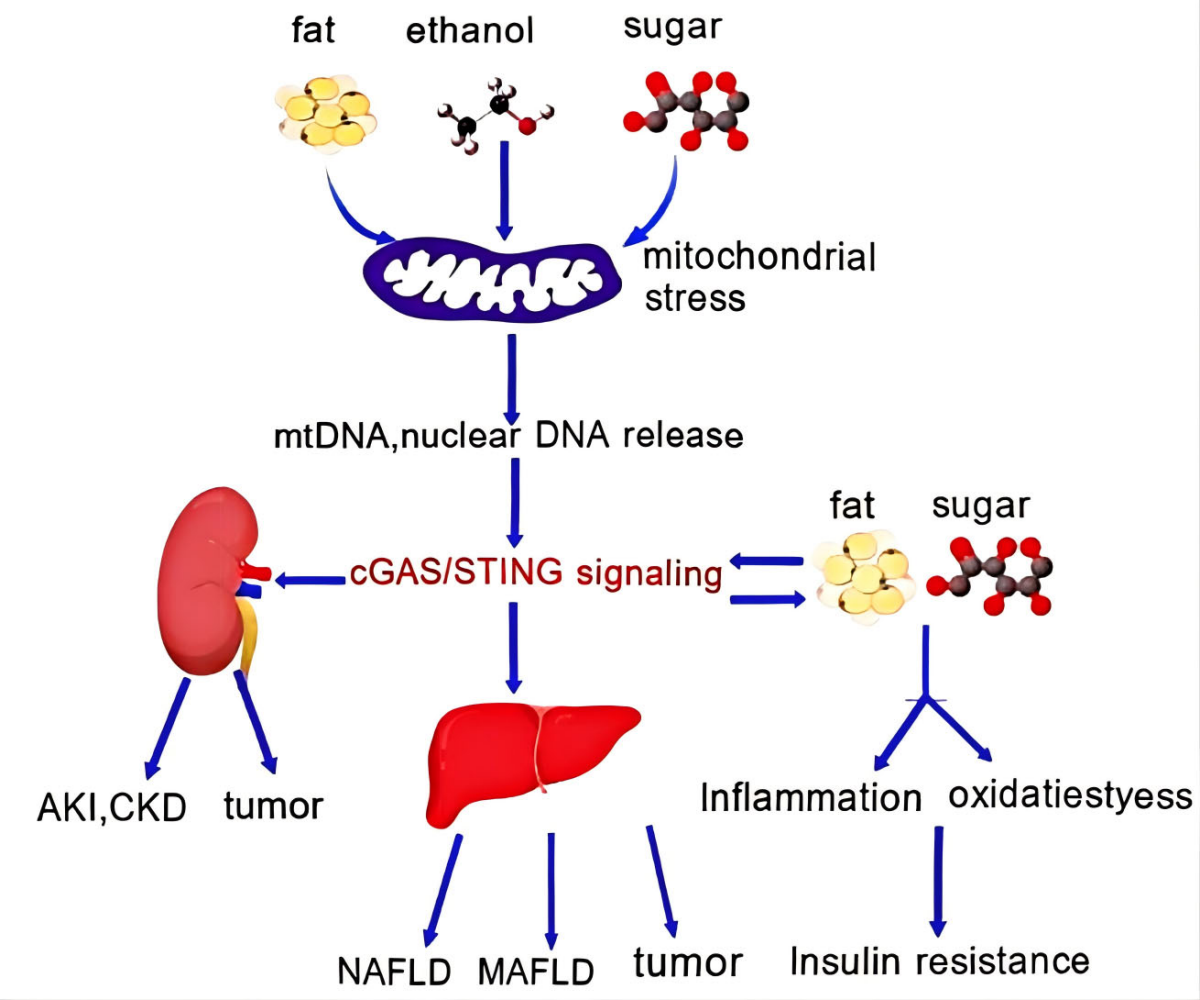
The cGAS stimulation pathways in metabolism and kidney disease. In metabolic tissues such as adipose tissue and liver, high-fat and high sugar diet or alcohol induces activation of the CGAS stimulation pathway. In adipose tissue, the activated CGAS spike pathway promotes inflammation, inhibits thermogenesis, leading to obesity and insulin resistance in the body. In the liver, CGAS nail activation is associated with the development of ALD, NAF, and Nash. In the kidneys, the activated CGAS nail pathway contributes to AKI, MAFLD, and tumors.

As a highly metabolic organ rich in mitochondria, the kidney relies on mitochondrial integrity; impaired mitochondrial function and tubular inflammation are established pathogenic mechanisms driving acute kidney injury (AKI) and subsequent CKD progression. Some studies have confirmed (102) that disulfide bond A oxidoreductase-like protein (DsbA-L) mitigates mitochondrial stress-induced mtDNA release and cGAS-STING pathway activation in adipose tissue, thereby alleviating high-glucose-induced renal tubular damage and preventing ectopic fat accumulation in diabetic nephropathy and fat-related kidney injury (103, 104). Additionally, the cGAS-cGAMP-STING axis has been shown to contribute to the development of AKI, immune-related kidney diseases, and renal tumors by recognizing self-DNA and initiating sterile inflammatory responses (105). (**Figure 2**).

### 2.2 The STING/NF-κB pathway

The STING and NF-κB signaling pathways are crucial in the pathogenesis of MAFLD (106, 107). Recent studies have shown that STING triggers downstream signals and activates the NF-κB pathway through non-canonical mechanisms, which exacerbates inflammatory responses and promotes hepatic lipid accumulation and fibrosis (108, 109).

**Figure 3:**
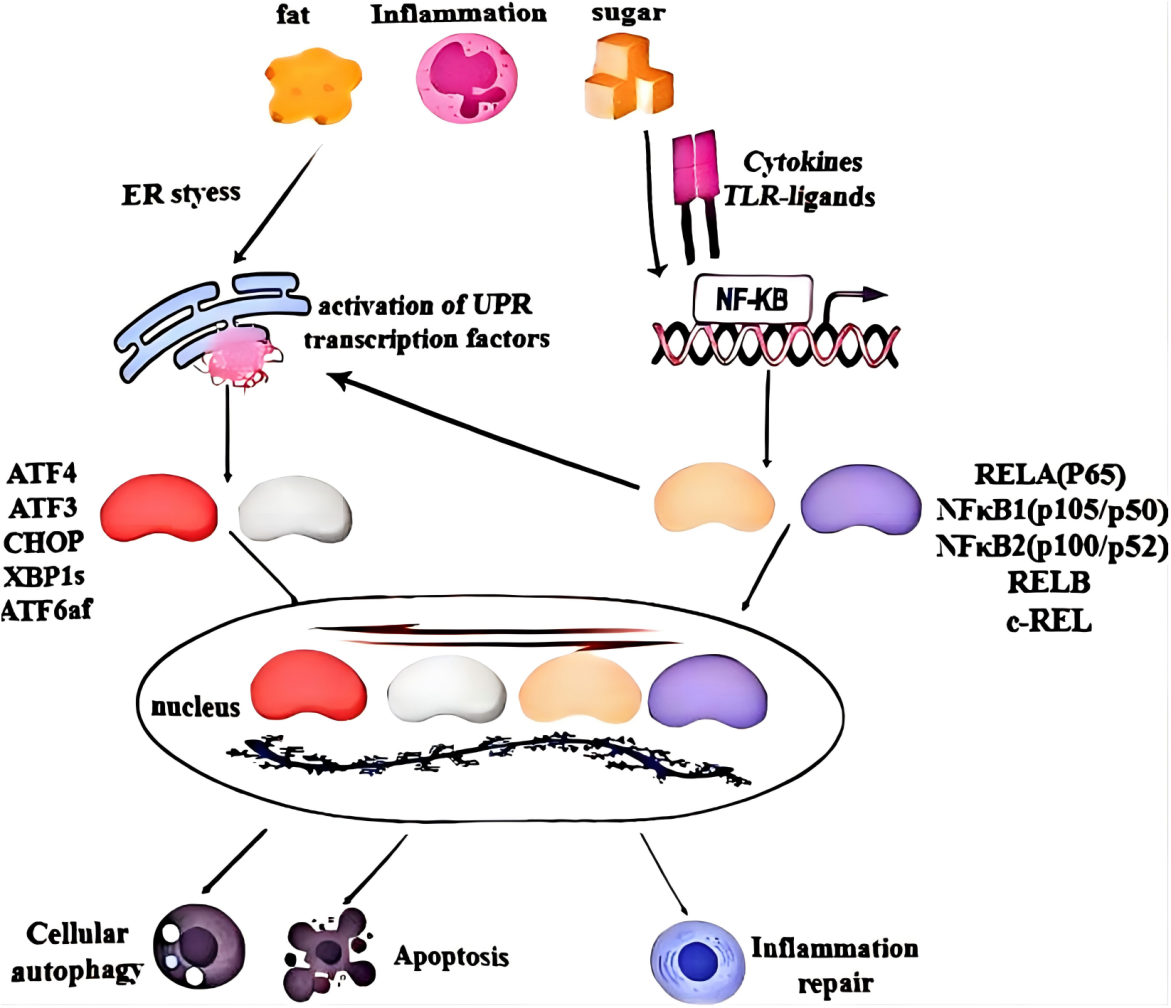
Integration of UPR and NF-κB signaling at chromatin. UPR and NF-κB pathways converge within the nucleus through ten major transcription factors. The combinatorial occupancy of numerous genomic regions (enhancers and promoters) coordinates the transcriptional activation or repression of hundreds of genes that collectively determine the balance between metabolic and inflammatory phenotypes and the extent of apoptosis and autophagy or repair of cell damage and survival.

New evidence reveals that the UPR and NF-κB pathways converge in the nucleus through ten key transcription factors (TFs): activating transcription factor 4(ATF4), ATF3, CCAAT/enhancer-binding protein (CEBP) homologous protein (CHOP), X-box binding protein (XBP) 1, ATF6α, and five NF-κB subunits. These TFs collectively bind to numerous genomic regions (enhancers and promoters), coordinating the transcriptional activation or repression of hundreds of genes. This genomic regulation determines the balance between metabolic and inflammatory phenotypes, as well as the outcomes of apoptosis, autophagy, damaged cell repair, and cell survival (110). (**Figure 3**).

NF-κB plays a pivotal role in regulating metabolic hormones (109), influencing major pathways such as glycation, triglyceride homeostasis, and lipogenesis. This transcription factor modulates the expression of key hormones like insulin and glucagon, which are essential for maintaining glucose levels and lipid metabolism (111). However, dysregulated NF-κB activation can disrupt hormonal balance, affecting adipocyte function and insulin signaling to impair lipid metabolism and energy homeostasis. Experimental models (112) demonstrate that mild hepatic NF-κB activation via IKβ regulation increases triglyceride levels and lipogenesis, while disrupting hypothalamic insulin and leptin signaling, leading to IR, energy imbalance, and obesity (113). These effects underlie the pathway’s role in triggering metabolic disorders and associated diseases (114, 115).

Chronic inflammation and fibrosis are central to metabolic-associated fatty liver pathogenesis, driving disease progression through inflammatory mechanisms that permeate all stages of MAFLD (116). NF-κB activation promotes the production of pro-inflammatory factors like TNF-α and IL-1β, amplifying the inflammatory cascade (117). This exacerbates hepatic metabolic dysfunction, induces hepatocyte damage, and accelerates the transition from MAFLD to non-alcoholic steatohepatitis (NASH) and fibrosis. Meanwhile, activated hepatic stellate cells (HSCs) transdifferentiate into myofibroblasts. These cells secrete collagen and extracellular matrix components, ultimately leading to hepatic fibrosis (118). Established fibrosis further impairs hepatic function and may progress to cirrhosis.

**Figure 4:**
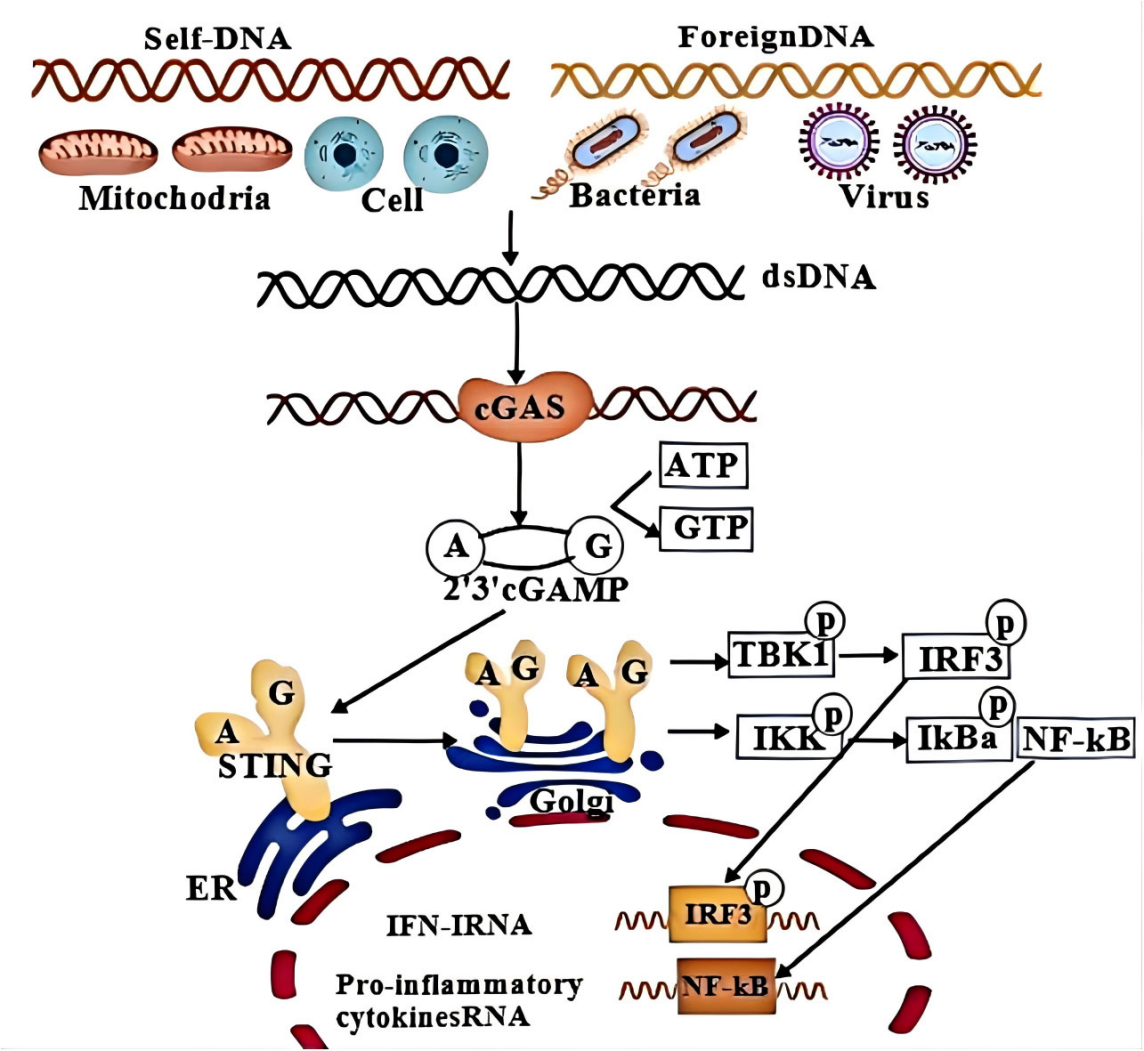
Molecular mechanism of cGAS-STING- NF-κB signaling. cGAS senses aberrant or mislocalized dsDNA to generate cGAMP, binding to STING to induce the relocation of STING to Golgi apparatus and trigger activation of TBK1 to result in phosphorylation of both STING and IRF3 transcription factor. In addition, IFN-I is initiated ^upon^ translocation of IRF3 into the nucleus, which subsequently induces various IFN-stimulated genes. At the same time, STING has ability to enlist IKK, which then triggers phosphorylation of the NF-κB inhibitor, IkBa, speeding up movement of NF-κB into the nucleus, which in turn enhances the production of certain inflammatory cytokines.

In summary, cGAS detects abnormal or mislocalized dsDNA, initiating activation and production of the second messenger cGAMP (57, 58). Binding to STING triggers its translocation to the Golgi apparatus, where TBK1 is activated, phosphorylating both STING and IRF3 (59). Phosphorylated IRF3 translocates to the nucleus, initiating type I interferon (IFN-I) production and downstream IFN-stimulated gene expression. Simultaneously, STING engages the IκB kinase (IKK) complex to phosphorylate the NF-κB inhibitor IκBα, facilitating NF-κB nuclear translocation and enhancing the transcription of pro-inflammatory cytokines (108, 109). This coordinated signaling amplifies both innate immune and inflammatory responses, driving MAFLD progression. (**Figure 4**).

### 2.3 Feedback of the cGAS/STING/NF-κB signaling pathway

In MAFLD, the interplay between cGAS, STING, and NF-κB creates a vicious cycle. Obesity, inflammation, and hepatic fat accumulation trigger cGAS activation, which subsequently activates STING. Activated STING then initiates NF-κB signaling, driving a robust inflammatory response that can further amplify STING activation.

**Figure 5:**
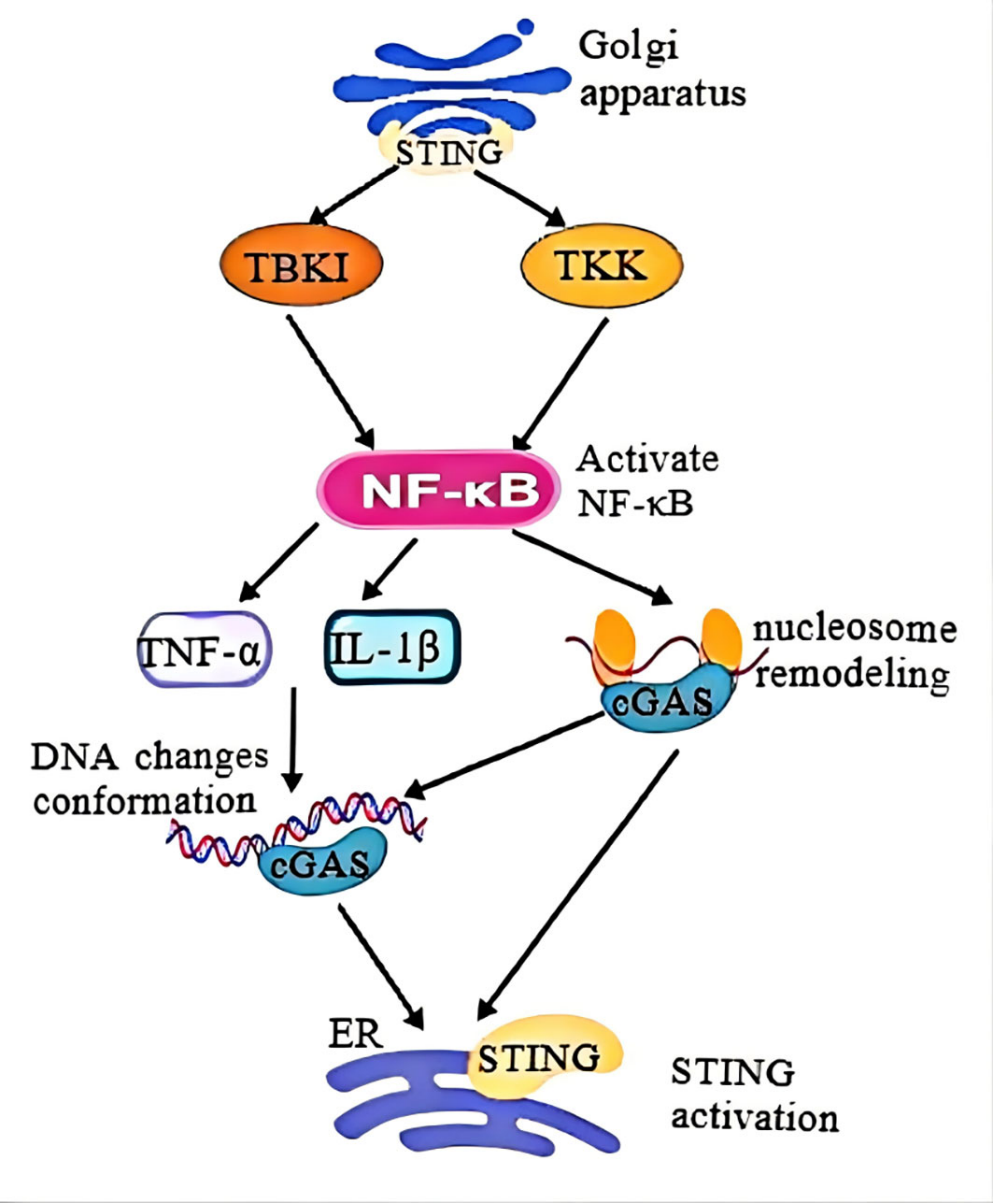
NF-κB is activated into the nucleus, regulates the release of inflammatory factors such as TNF-α and IL-1β, and causes changes in DNA conformation, thus initiating the innate immune response. NF-κB can also induce nucleosome remodeling, in which cGAS is released from nucleosome isolation, allowing it to function in response to dsDNA, thereby activating the CGAS-STING signaling pathway.

This crosstalk forms a feedback loop critical for liver injury and MAFLD progression (119). (**Figure 5**).

Upon activation, NF-κB translocates to the nucleus, where it regulates the expression of inflammation-related genes and triggers inflammatory responses. This process is accompanied by DNA conformational changes that initiate canonical innate immune responses (120). During Toll-like receptor 4 signaling, NF-κB coordinates inducible nucleosome remodeling, activating enhancers and promoters within open chromatin regions (121). By remodeling nucleosomes, cGAS is released from nucleosomal sequestration, enabling its interaction with cytosolic dsDNA to activate the cGAS-STING pathway (46). Open chromatin structures also enhance cGAS-DNA interactions, facilitating pathway activation (122, 123).

**Figure 6:**
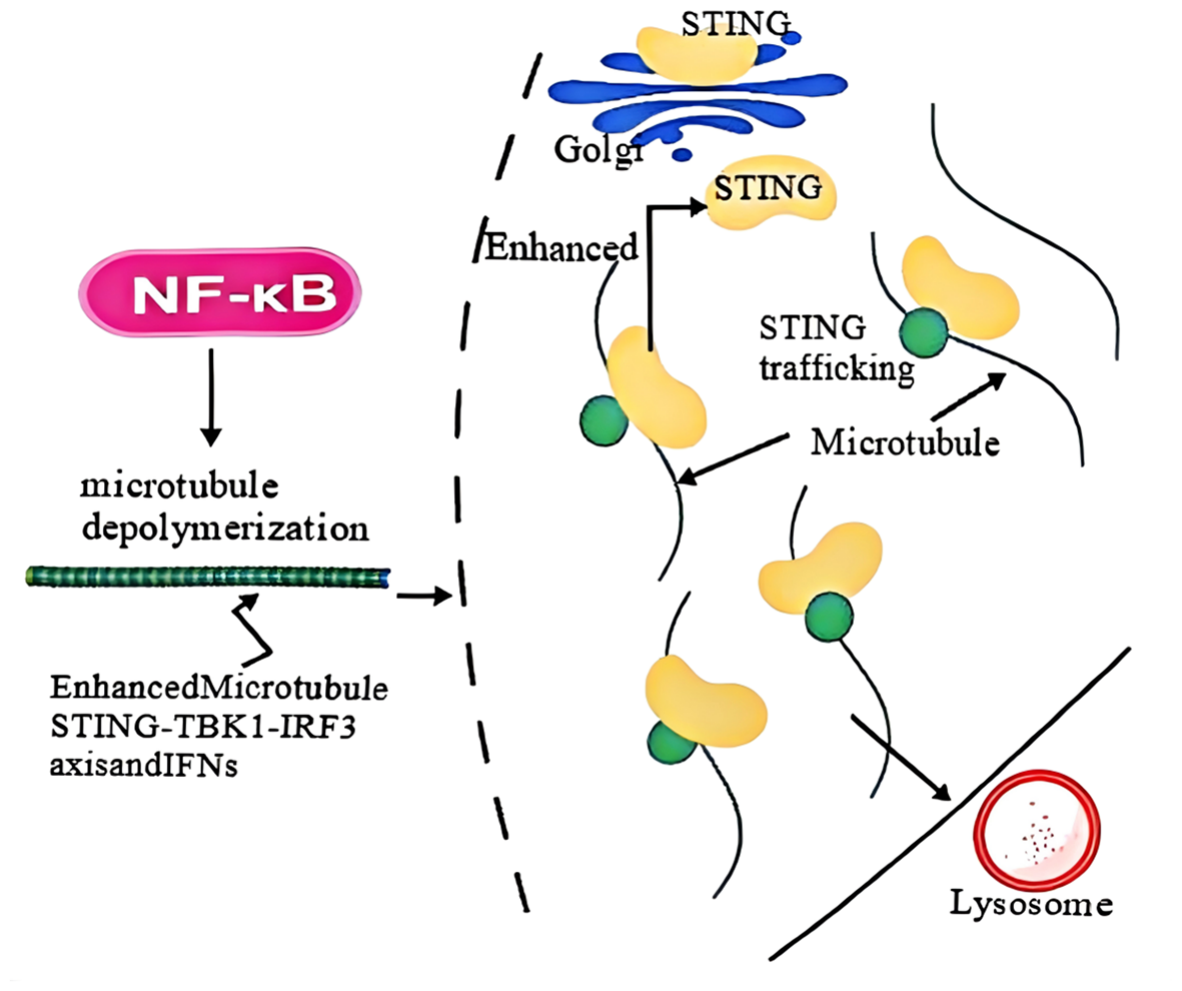
Activation of the NF-κB pathway enhances STING-mediated immune response. NF-κB activation prolongs and ^increases^ STING signaling by inhibiting microtubule-mediated transport of STING from austenite to lysosome, thereby blocking STING degradation. This enhances STING-mediated interferon response and host antiviral defense.

Research shows that STING relies on microtubules for intracellular transport. NF-κB pathway activation induces microtubule disassembly, inhibiting STING transport to lysosomes for degradation and sustaining prolonged STING activation. This synergy between NF-κB and STING amplifies interferon responses. Additionally, gain-of-function STING mutations disrupt microtubule binding, causing abnormal STING trafficking and ligand-independent autoactivation. Thus, NF-κB enhances STING signaling by regulating microtubule-mediated transport, perpetuating inflammatory cascades (124). (**Figure 6**).

The dysregulated cyclic activation of this signaling pathway may promote further liver inflammation and cellular damage, leading to progressive exacerbation of hepatic inflammation in MAFLD patients and potentially driving the development of liver fibrosis and even hepatocellular carcinoma (125). Studies also indicate that cGAS/STING/NF-κB pathway activation induces inflammation and IR (102), with IR representing a central driver of MAFLD pathogenesis that arises from interactions across multiple signaling networks (38, 64).

In summary, within metabolic-associated fatty liver diseases, the cGAS/STING and NF-κB pathways exhibit intricate crosstalk: cGAS/STING activation triggers NF-κB signaling, driving the production of pro-inflammatory cytokines such as TNF-α and IL-6. These cytokines intensify hepatic inflammation, disrupt glucose and lipid metabolism, induce hepatocyte fat accumulation and IR, and collectively accelerate fatty liver progression.

## 3 Treatment strategies for intervening in the cGAS/STING/NF-κB signaling pathway in MAFLD

Given the critical role of the cGAS/STING/NF-κB signaling pathway in MAFLD, developing drugs targeting this axis offers promise as a novel therapeutic strategy. Current treatment approaches focusing on this pathway primarily include the following aspects:

### 3.1 Small-molecule inhibitors

Blocking specific steps in the cGAS-STING cascade may alleviate inflammation. Targeted small-molecule inhibitors of cGAS or STING effectively dampen inflammatory responses (59). These agents counteract MAFLD progression by reducing hepatic inflammation and damage—for example, by blocking cGAS-DNA binding or interfering with STING downstream signaling.

DNA Release Regulation: PPARα may modulate cGAS-STING transduction via mitochondrial DNA (mtDNA) release, suggesting PPAR pathway modulation could regulate cGAS activation (126). Tanreqing has been shown to inhibit mtDNA release and STING-mediated signaling *in vitro (* 127).

RU.521 is a potent and selective cGAS inhibitor capable of potently suppressing cGAS-mediated interferon upregulation, thereby inhibiting the onset of inflammation. As a cGAS inhibitor, RU.521 reduces downstream STING-induced IFN and pyroptosis (128).

STING Inhibition: STING-specific inhibitors like C-170 and C-176 block cGAMP binding to STING (129), while H-151 inhibits STING/NF-κB signaling in immune cells (130).Recent studies have shown that the LXR/RXR agonist UAB116, whose known functions include regulating cholesterol metabolism, lipid transport, and immune response, can upregulate TRIM29 (131). As a widely existing ubiquitin E3 ligase, TRIM29 is a key negative regulator of the STING signaling pathway. It can inhibit inflammatory responses and oxidative stress, as well as negatively regulate antiviral immunity by degrading the downstream molecule STING (132, 133).

In addition, TRIM29 can alleviate endoplasmic reticulum stress by regulating the activity of PERK, thereby reducing hepatocyte damage in metabolic-associated fatty liver disease and improving metabolic outcomes. As a core regulatory factor of the UPR, PERK mainly functions to sense endoplasmic reticulum stress and helps cells restore homeostasis by regulating protein synthesis and degradation processes (134, 135).

It should be noted that TRIM29 promotes K48-linked ubiquitination, which can lead to the degradation of NOD-like Receptor Family Pyrin Domain Containing 6(NLRP6) and NOD-like Receptor Family Pyrin Domain Containing 9b (NLRP9b). This degradation reduces the secretion of IFN-λ and IL-18 by intestinal epithelial cells (IECs), impairs the inhibitory effect of NLRP6 on other inflammatory pathways (such as the NF-κB pathway and TLR signaling pathway), and ultimately induces intestinal inflammation (136).

Furthermore, the reduction in IL-18 (an effector protein of NLRP6) disrupts the negative regulation of the intestinal microbiota, allowing TLR4 and TLR9 agonists to enter the portal vein. This leads to increased TNF-α expression in the liver, exacerbates hepatic steatosis and inflammation, and promotes the progression of NAFLD/NASH (137). Therefore, the exploration of drugs that bypass TRIM29 and act on NLRP6—such as pioglitazone, which can upregulate the expression of NLRP6 (138)—is of great significance. The combined action of such drugs with TRIM29 may play a positive role in the comprehensive improvement of MAFLD.

Although current research on directly targeting TRIM29 for MAFLD treatment is still in its early stages, TRIM29 has potential effects by regulating mechanisms such as the STING pathway, immune microenvironment, DNA damage repair, and alleviation of endoplasmic reticulum stress. Its combined use with NLRP6 agonists may provide a new strategy for MAFLD treatment.

Notably, TRIM29 also exhibits a dual function of suppressing or promoting cancer in various types of cancer: it exerts a cancer-promoting effect in cancers such as lung cancer, colon cancer, nasopharyngeal carcinoma, esophageal cancer, and pancreatic cancer, while in liver cancer, it can reverse the resistance to lenvatinib (139–147).

### 3.2 Anti-inflammatory therapies

Pharmacological interventions targeting inflammation, such as glucocorticoids, downregulate NF-κB activation and reduce pro-inflammatory cytokine production. Clinical studies indicate these drugs can alleviate hepatic inflammation in NASH patients (31, 148).

Signaling Pathway Modulation: Insulin promotes anti-inflammatory macrophage polarization and inhibits NF-κB expression (149, 150), while tofacitinib and aspirin suppress JAK-STAT and NF-κB pathways, respectively, mitigating type 2 diabetes progression (151). Ferulic acid prevents M1 macrophage polarization in diabetes-related inflammation by inhibiting TLR4/IL-6-mediated NF-κB/JAK-STAT signaling (AGE-RAGE interactions) (152).

Metal Ion Regulation: Zinc ion (Zn²^+^) chelator TPEN inhibits intracellular cGAS activation, highlighting metal ions as potential regulators of cGAS-STING-mediated inflammation (153).

### 3.3 Nutritional and lifestyle interventions

Dietary adjustments and increased physical activity can partially regulate the cGAS/STING/NF-κB pathway, improving hepatic metabolic status (125, 154). Reducing high-fat intake, for example, decreases cGAS/STING activation and alleviates fatty liver symptoms.

### 3.4 Pathway dual roles and precision medicine

While the cGAS/STING/NF-κB axis promotes inflammation and metabolic dysfunction in MAFLD, it may exhibit protective effects during infections or immune responses by enhancing pathogen resistance (155). Understanding its context-dependent dual functions is critical for developing targeted therapies. Advances in genomics and proteomics will enable personalized treatment strategies by elucidating interindividual variations in pathway activity (124).

### 3.5 Novel drug development

Future research is likely to yield more clinical-specific cGAS/STING/NF-κB inhibitors. Combined therapies targeting multiple signaling pathways (such as nanomedicines for regulating cGAS-STING and drugs targeting TRIM29 for treatment) are expected to improve therapeutic efficacy (156).

### 3.6 Multidisciplinary collaboration

Treating MAFLD requires integrated efforts from endocrinology, gastroenterology, and immunology disciplines (52, 157). Interdisciplinary research will deepen understanding of the pathway’s role in metabolic diseases, accelerating the development of innovative therapeutic strategies.

## 4 Conclusion

The cGAS/STING/NF-κB signaling pathway plays a crucial role in the pathogenesis and progression of MAFLD. Activation of this pathway not only triggers hepatic inflammatory responses and IR but also regulates lipid metabolism, thereby promoting the development of hepatic fibrosis and cirrhosis. Therefore, targeting the cGAS/STING/NF-κB signaling pathway has become a highly promising novel therapeutic strategy for MAFLD.

However, due to the complexity of the immune regulatory network, the molecular differences and sources of factors (such as hyperglycemia, lipid accumulation, and viruses) that trigger abnormal DNA release have not been fully elucidated. Moreover, there may be differences in regulatory mechanisms across different stages of MAFLD—including simple fatty liver, steatohepatitis, hepatic fibrosis, and cirrhosis—as well as variations in the components of metabolic abnormalities among affected individuals. These aspects require in-depth analysis. In the future, efforts should be made to deepen the exploration of molecular mechanisms such as metabolomics and proteomics, and accurately identify the upstream triggers of cGAS activation. This will provide support for the individualized treatment of MAFLD, facilitate the development of more effective drugs and intervention strategies, and ultimately bring new hope to the clinical management of MAFLD.
